# The Care of the Hands

**Published:** 1907-05-18

**Authors:** 


### Resident Medical Officers' Department.

### THE CARE OF THE HANDS.

We published recently in this section of The Hospital a short article on the health of resident medical officers. A few practical suggestions were made on the subjects of food, fresh air, exercise, sleep, and recreation. The article did not profess to do more than indicate the main points which a house surgeon should keep before him in the care of his own health. This subject is, however, of such importance, and is so frequently neglected by resident medical officers and ignored by hospital house committees, that we propose to refer to it again from time to time, as opportunities present themselves.

This week we have chosen the subject of the care of the house surgeon's hands. To the non-medical ear this may sound at first like an absurd refinement, bordering on the luxurious and the æsthetic. But to those engaged in the practice of surgery, or in the making of vaginal or post-mortem examinations, it is a matter of the first importance, affecting not only health, but often also life itself.

Septic fingers (or whitlows) and septic throats (or hospital sore throats) are the scourges of hospital life. House surgeons, nurses, and dressers are constantly being incapacitated by them. With sore throats, their nature, prevention, and treatment, we do not propose to concern ourselves at present, beyond pointing out that, although they are accompanied with great misery and prostration, they seldom involve danger to life or even leave permanent after-effects.

Septic fingers, on the other hand, however trivial they may appear at first, are always full of the most dangerous possibilities. Mutilations, deformities, and disfigurements, to say nothing of fatal septicæmias, are frequent consequences of what seem in the beginning to be but mild infections of the nail-bed or finger-pulp. Constitutions weakened by hospital work offer feeble resistance to septic infections, and poisoned fingers which would heal up in a few days in the country, usually linger on for weeks in hospital. Even if they do not lead to cellulitis or involvement of the tendon sheaths, or to septicæmia, they interfere with the medical officer's work, and in spite of every precaution may give rise to infection of his patients' operation-wounds. A whitlow is a septic focus—a continual menace to the house surgeon himself, and to every patient under his care.

What we wish to point out is that septic fingers are avoidable evils, and that their prophylaxis can be summed up in a phrase—care of the hands. Regular and systematic attention to his hands, and especially to the skin around the nails, is quite possible to the busiest of house surgeons.

The constant scrubbing of the hands and nails with stiff nail-brushes, and the free use of irritating antiseptics, which form the routine method of skin sterilisation for operations and ward dressings, are very trying to the skin of the house surgeon's hands, particularly in cold dry weather. Unless he takes precautions, a few days of hard surgical work lead to innumerable tiny cracks and "hang-dogs" on his fingers; and if his skin is unusually sensitive his hands will soon be affected with an intractable form of dermatitis. In this condition he is peculiarly liable to staphylococcal or streptococcal infection, and a septic finger, with perhaps the loss of a nail or the appearance of an axillary abscess, is only too likely to result. Should he make a vaginal examination on a syphilitic subject or conduct a post-mortem without gloves when his hands are in this state the consequences may, of course, be even more disastrous.

What precautions, then, should the house surgeon take? After washing his hands at the conclusion of each afternoon's operations and each morning's dressings he should rub in, until absorbed, some soothing or stimulating preparation of glycerine, such as "Glycola," or glycerine and honey jelly, or glycerine and red wash, or glycerine and eau-de-Cologne—whichever he finds most suitable—and should clip off every little tag of skin, and press down with a blunt instrument the free margin of skin at the base of each nail. If the fingers are still cracked and sore when he goes to bed at night he should wash them in hot water, and, when dry, rub into them some lanoline and castor oil or Hazeline cream; and if the condition is advanced he should sleep in chamois leather gloves. Before the next day's work he should paint over every abrasion or fissure a film of "New Skin" or collodion, or he should wear aseptic india-rubber finger-stalls or gloves.

All this sounds very tedious and elaborate; but in practice it is easy to carry out, and if the preventive measures with glycerine preparations are regularly adopted, the disagreeable greasy applications are seldom necessary. "Glycola," etc., is kept on the washhand-stands in the wards and theatres of most hospitals, and its use soon becomes a habit, as pleasant as it is necessary.

If a scratch or cut occurs at a septic operation or post-mortem examination, it should be cleansed at once with antiseptics and covered with waterproof protective, or, better, enveloped in a large hot boracic fomentation for a few hours, and then protected.

Similarly, if a finger not previously known to be abrased becomes painful and hot soon after an occasion of this sort, it should at once be treated with wet antiseptic applications, as hot as can be borne. By this means the most virulent infections can often be aborted. Thus, soaking the hand in hot biniodide of mercury solution (1-1,000) is excellent early treatment for septic fingers. Should, however, the arm show signs of invasion, in spite of these measures, not a moment should be lost in seeking the aid of one of the senior surgical staff, for the worst forms of septic infection of the hand spread with the most appalling rapidity, and require immediate operation if life is to be saved.

